# Avascular Necrosis With Complete Fragmentation and Collapse of the Femoral Head Treated With Cementless Total Hip Arthroplasty: A Case Report

**DOI:** 10.7759/cureus.86976

**Published:** 2025-06-29

**Authors:** Tsamara Roberts, Amanda Penn, Logan Hanson

**Affiliations:** 1 College of Osteopathic Medicine, Lake Erie College of Osteopathic Medicine, Bradenton, USA; 2 Department of Orthopedic Surgery, Bond Clinic, Winter Haven, USA

**Keywords:** avascular necrosis, case report, cementless, femoral head, total hip arthroplasty

## Abstract

Avascular necrosis (AVN) of the femoral head results from disrupted blood flow that leads to bone tissue death. Nontraumatic risk factors include long-term corticosteroid use, excessive alcohol consumption, autoimmune diseases, and hematological disorders. Without an adequate blood supply, osteocytes die, causing microfractures that lead to structural collapse of the femoral head. Disruption of joint biomechanics causes hip or groin pain and limited range of motion (ROM). This case report presents a 56-year-old male patient with severe left hip pain and progressive functional decline over nine months. Imaging confirmed stage IV AVN with complete femoral head collapse. The patient failed initial conservative measures and underwent a cementless total hip arthroplasty (THA) augmented with peripheral screws in the acetabular cup. Post-operative recovery was unremarkable, with reported improvement of pain and increased hip motion at the six-week follow-up. This case highlights the importance of early diagnosis and treatment in advanced avascular necrosis of the femoral head. It also demonstrates the effectiveness of cementless total hip arthroplasty in providing good clinical outcomes.

## Introduction

Avascular necrosis (AVN) of the femoral head is a condition where bone tissue dies due to a lack of blood flow. Compromised blood flow can have traumatic and nontraumatic etiologies. Risk factors for nontraumatic causes include long-term corticosteroid use, excessive alcohol consumption, autoimmune diseases, and hematological disorders [1]. Without adequate blood supply, osteocytes die, leading to the development of microfractures, with the subchondral bone being particularly vulnerable. Over time, the accumulation of these microfractures results in loss of structural support and disruption of normal hip joint biomechanics [2]. Patients with AVN typically present clinically with severe hip or groin pain with a limited range of motion (ROM).

The treatment of AVN is geared toward reducing pain and restoring hip function. Treatment is based on disease severity, which can be categorized using the Ficat and Arlet classification system. The Ficat and Arlet classification is a staging system that uses radiographic images to identify the progression of avascular necrosis of the femoral head. It ranges from stage I, where there are no radiographic changes identified, to stage IV, where joint collapse and osteoarthritis are present. The early stages of the disease can be managed non-operatively with joint-preserving treatments. However, for advanced disease, particularly when there is collapse of the femoral head, total hip arthroplasty (THA) is the treatment of choice [3].

A total hip arthroplasty is a surgical procedure in which a damaged femoral head and acetabulum are removed and replaced with artificial implants. Either a cemented or cementless technique can be used to secure the implants to the patient's bones. In a cemented total hip arthroplasty, polymethylmethacrylate, a synthetic acrylic polymer commonly used as bone cement, is used to anchor the implants to the bone [4]. On the other hand, the implants used in a cementless total hip arthroplasty are designed with porous and textured material to encourage the surrounding bone to grow into the implants in a process called osseointegration [5]. In patients with AVN, cementless THA has been shown to provide favorable long-term outcomes with significant improvement in pain and increased range of motion [6].

This case report describes a patient with severe AVN of the left hip, characterized by complete fragmentation and collapse of the femoral head, who underwent a cementless total hip arthroplasty augmented with peripheral screws and dual mobility cup. The purpose of this case report is to illustrate the extent of disease progression associated with avascular necrosis, portraying that it can result in total collapse of the femoral head, allowing for manual removal. This case report also aims to highlight the importance of using intra-operative findings to guide medical decision-making. The decision to use peripheral screws and a dual mobility cup in this case reflects a strategic effort to increase implant stability and longevity to provide the best surgical outcome.

## Case presentation

The patient is a 56-year-old man with no pertinent past medical history who presented with significant pain and decreased motion about his left hip. He stated that the pain initially started nine months ago and has increasingly progressed since its onset. At the time of presentation, he was unable to walk short distances without experiencing debilitating pain. He reports that these symptoms were beginning to interfere with his quality of life. Physical examination of the left hip demonstrated normal passive range of motion with pain on internal rotation. There were no local masses or tenderness observed.

**Figure 1:**
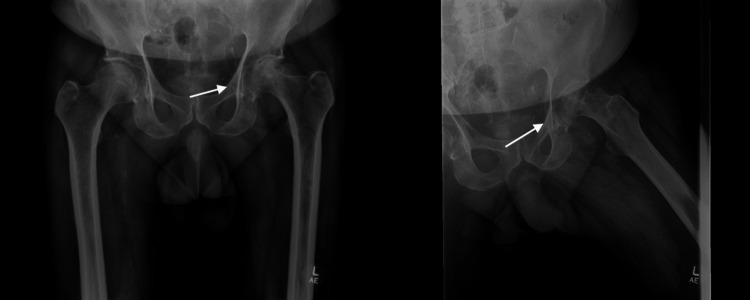
Anteroposterior and lateral X-ray of the pelvis demonstrating significant degenerative changes with collapse of the femoral head

**Figure 2:**
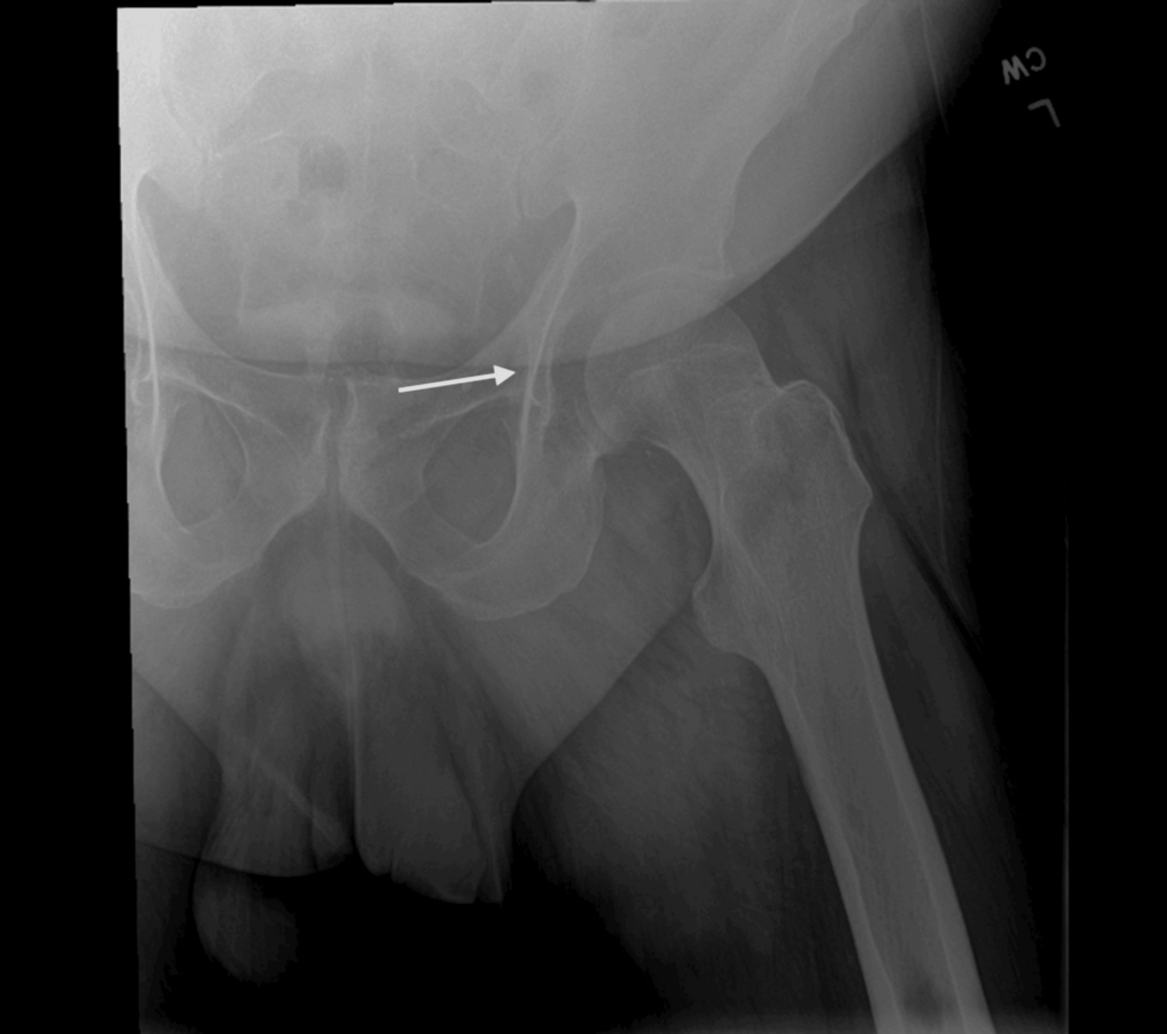
Lateral X-ray of the hip nine months prior to seeking orthopedic evaluation

X-rays of the left hip at this time revealed significant degenerative changes with collapse of the femoral head (Figure 1). This reflects a notable progression of disease when compared to earlier imaging at symptom onset, which showed only mild osteoarthritis (Figure 2). The patient received a steroid injection into the left hip two weeks prior to presentation that did not alleviate his symptoms. The progression of his symptoms and radiographic evidence of structural joint compromise directly correlate with worsening disease. The decision was made to proceed with total hip arthroplasty due to clinical deterioration despite conservative management.

**Figure 3:**
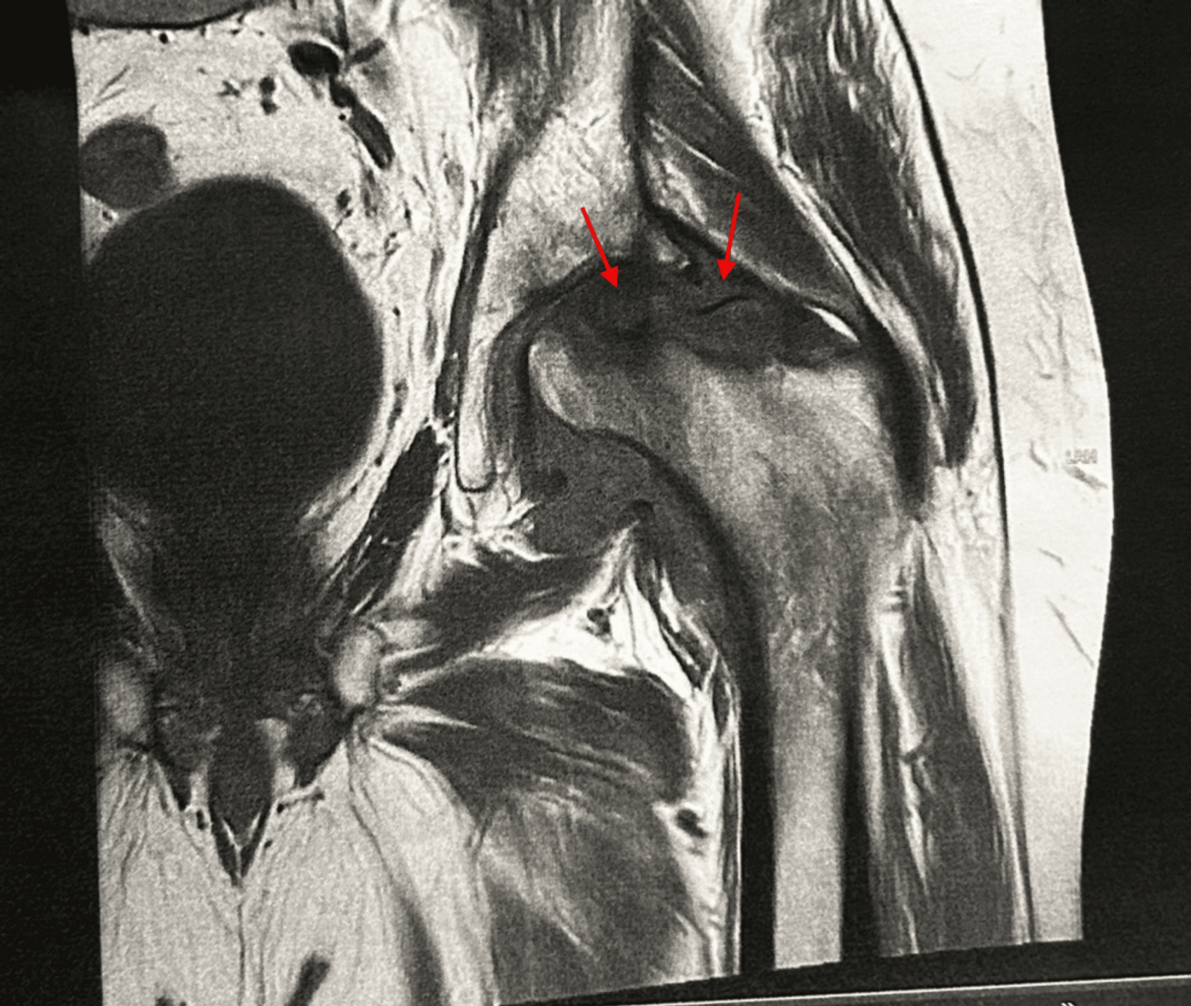
Magnetic resonance imaging of the left hip without contrast demonstrating crescentic fracture line

Prior to the surgery, a T2-weighted magnetic resonance imaging (MRI) was ordered to rule out any additional intra-articular pathology. MRI of the left hip without contrast identified a large crescentic fracture line that extended through the superior femoral head with a sclerotic fragment in situ involving most of the weight-bearing articular surface measuring up to 2.70 centimeters transverse, 3.37 centimeters anteroposterior, and 1.40 centimeters craniocaudal with extensive edema seen in the residual femoral head and neck (Figure 3). These findings are consistent with stage IV left hip avascular necrosis.

**Figure 4:**
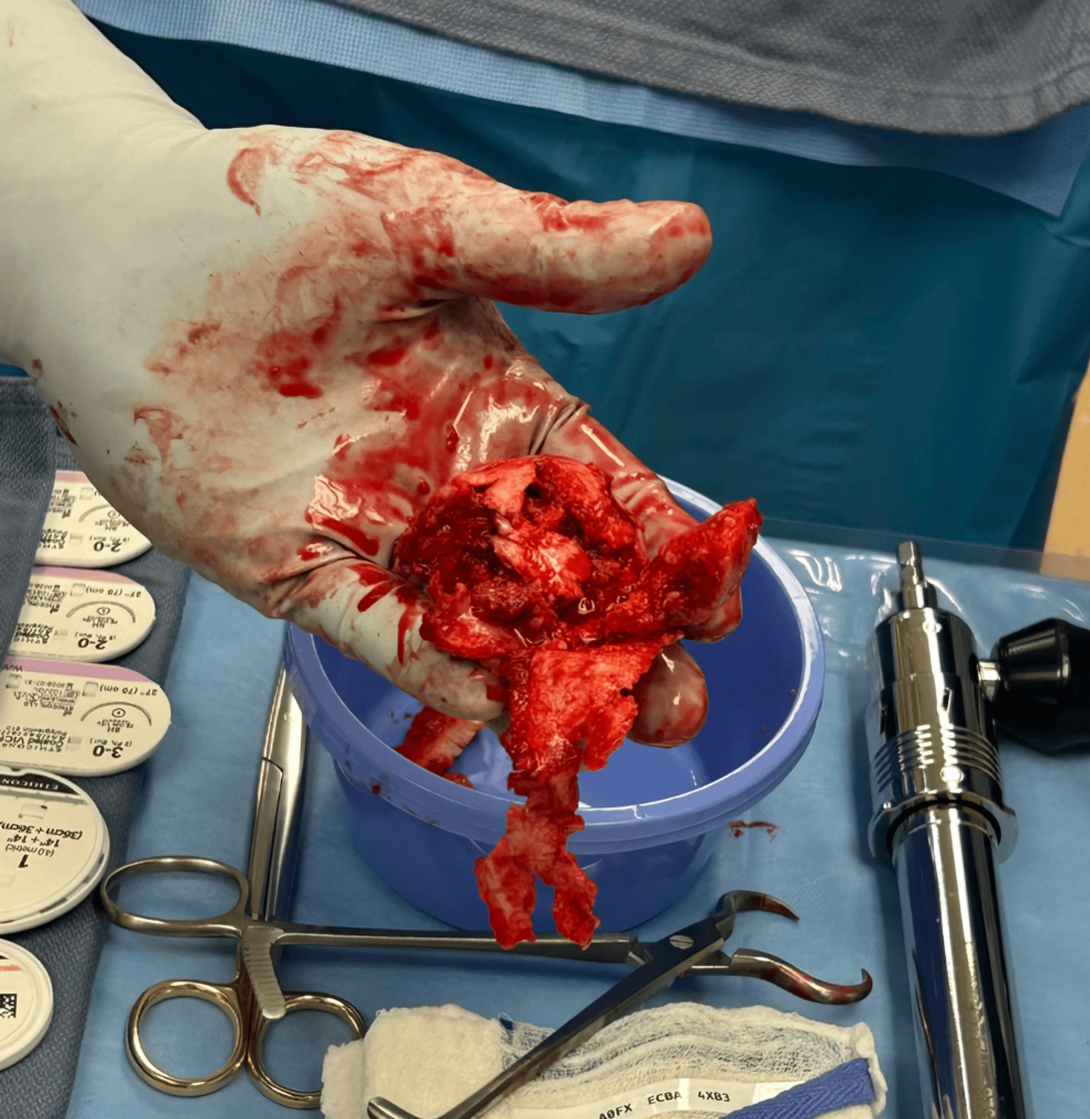
Left femoral head following manual removal

On the day of surgery, the patient was taken back into the operating room, and after induction of general anesthesia, he was positioned supine on the Hana table. An anterior approach was used with the incision marked 3 centimeters distal and 1 centimeter posterior to the anterior superior iliac spine (ASIS), curving laterally toward the anterior border of the femur, measuring approximately 12 centimeters. Superficial and deep dissection was performed. The anterior capsule was identified, and an anterior capsulotomy was performed. The patient had significant thickening of the anterior capsule. The femoral neck cut was made, and a second subcapital femoral neck cut was subsequently made with removal of the circumferential rim of sclerotic bone. After this occurred, the femoral head was located within the acetabulum and was completely fragmented and collapsed. The femoral head was then manually removed (Figure 4).

The patient had poor bone quality with extensive anterior acetabular wear. The acetabulum was sequentially reamed to a size 57 to accommodate a 58 cup. Once the acetabulum cup was placed, it was then augmented with three peripheral screws due to the acetabulum being extremely soft. The peripheral screws demonstrated good stability. A dual mobility liner was then impacted into place, and attention was turned to the femur. The femur was sequentially broached to size 14, and poor bone quality was noted in the femoral canal. Once the trial components appeared to be positioned appropriately, the size 14 micro taperloc stem was anchored into place. Following this step, a standard neutral dual mobility head was placed. A dual mobility bearing construct was utilized to enhance implant stability and reduce the risk of post-operative dislocation based on the intra-operative identification of diminished bone integrity. The hip was reduced, and final intra-operative radiographs were obtained. The wound was irrigated, and vancomycin powder was introduced. The tensor fascia lata (TFL) fascia was closed with a number 2 quill, the skin was approximated with 2-0 and 3-0 vicryl, and the subcuticular skin was approximated with 3-0 quill followed by application of surgical skin glue and Mepilex dressing.

**Figure 5:**
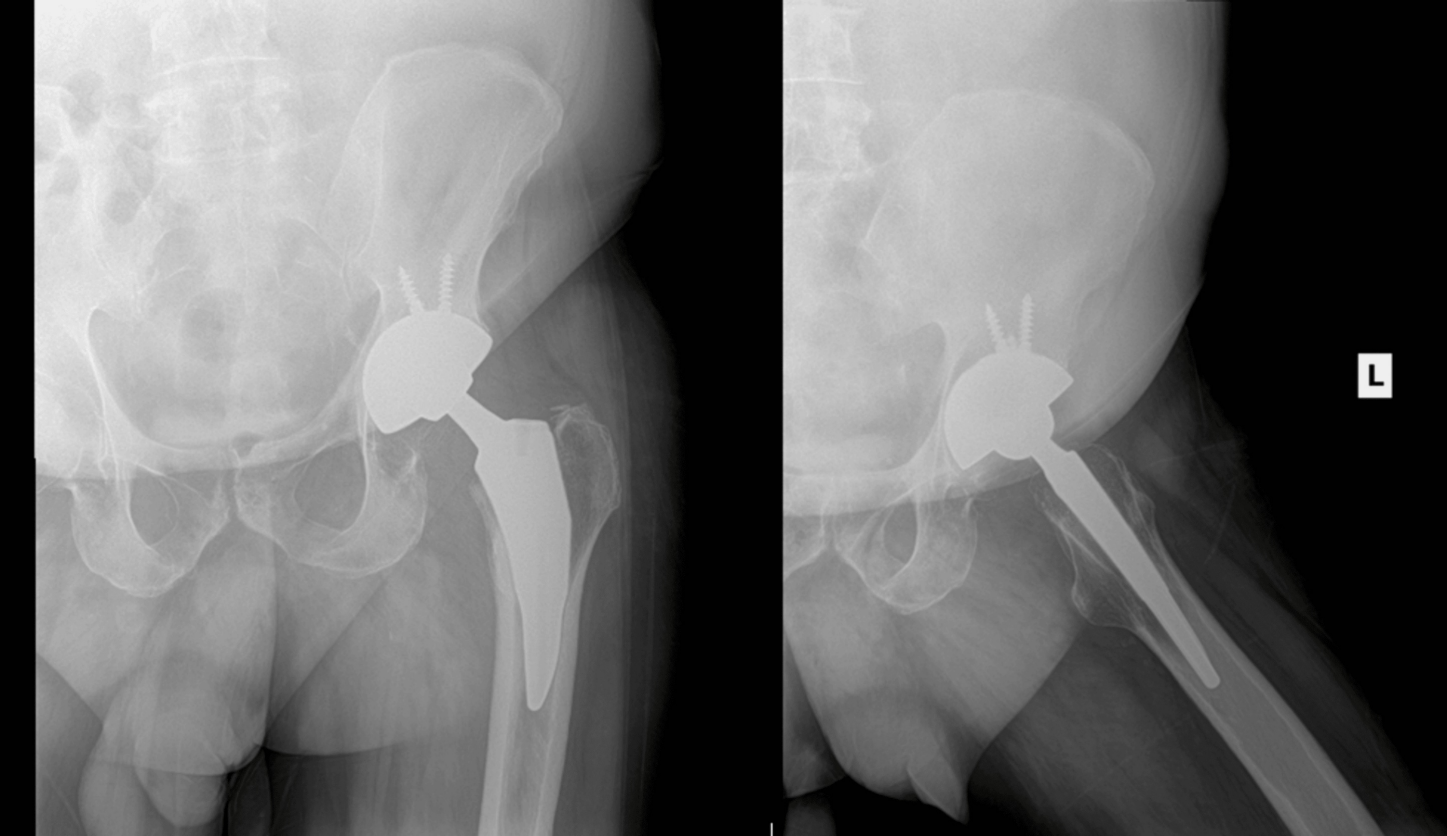
Anteroposterior and lateral X-ray of the left hip two weeks post-operatively demonstrating appropriate implant placement

The patient's immediate post-operative course was unremarkable. At a two-week post-operative visit, the patient was ambulating with crutches and reported minimal pain with no numbness or tingling. Physical examination showed the pelvis to be level and a well-healed incision with no signs of infection. The patient had passive ROM that showed normal flexion to greater than 90 degrees and full extension. Internal and external rotation of the left hip demonstrated minimal discomfort with a normal arc of motion. X-ray at this time revealed no signs of hardware disruption, fractures, or dislocation, with post-operative soft tissue changes (Figure 5). At his six-week post-operative visit, the patient continued to do well. He did report a mechanical fall one week prior to the visit but noted that his pain had improved and was similar to his pre-operative baseline. His physical examination was unchanged from the two-week follow-up, and X-ray at this time demonstrated left total hip arthroplasty with no signs of hardware disruption, fractures, or dislocation, and resolution of post-operative soft tissue changes. The patient will continue to be monitored with outpatient follow-up, with the expectation of a good clinical outcome.

## Discussion

The treatment of AVN is dependent on the progression of the disease state. The Ficat and Arlet classification system, summarized in Table 1, is a commonly used method to identify four stages of AVN based on radiographic features. In stage I, the femoral head appears normal on radiographic imaging. Stage II occurs when there are cystic lesions or osteosclerotic lesions with a normal contour of the femoral head without subchondral fractures. Stage III is characterized by subchondral collapse producing the classic crescent sign. Stage IV presents with complete collapse of the femoral head and decreased joint space with secondary acetabular changes [7].

**Table 1:** Ficat and Arlet classification of avascular necrosis of the femoral head

Stages	Radiographic findings
Stage I	Normal X-ray findings
Stage II	X-ray will show sclerosis, cyst, or osteopenia; femoral head is preserved
Stage III	X-ray will show crescent sign, femoral head begins to collapse, and joint space is preserved
Stage IV	X-ray will show flattened femoral head, joint space narrowing, acetabular involvement, and osteoarthritis

Stage I and II can be managed non-operatively using pharmacological interventions such as bisphosphonates, anticoagulants, statins, and vasodilators [8]. In addition to these pharmacological options, management of the early stages of AVN can incorporate biophysical modalities such as extracorporeal shock wave therapy, pulsed electromagnetic therapy, and hyperbaric oxygen. As the disease progresses, joint-preserving procedures such as core decompressions, vascularized bone grafts, and redirectional osteotomies have been proven to provide clinically beneficial results [9]. Hip-conserving procedures become ineffective when there are more than 2 millimeters of collapsed femoral head or if there are secondary degenerative changes. For stage IV AVN, the treatment of choice is a total hip arthroplasty.

There have been numerous reports demonstrating the positive clinical outcomes of THA in patients with advanced AVN. The use of cementless fixations, as in our patient, has increased the longevity of THA with a 10-year survival rate of approximately 92.5% of patients with AVN [10]. Peripheral screws were used due to intra-operative identification of poor bone quality to enhance stability. Securing the acetabular component with peripheral screws has been shown to reduce the risk of aseptic loosening and provide long-term durability [11]. Dual mobility liners were also used to provide additional security. They are used to allow for a greater range of motion with the goal of reducing the risk of dislocation [12].

Not only are post-operative outcomes influenced by patient and procedural factors, but they may also be impacted by institutional and larger environmental elements. Non-municipal water sources have been shown to increase the risk of surgical site infections, emphasizing the importance of a sterile operating room environment [13]. In addition, the timing of surgical intervention is a contributing factor to surgical outcomes, especially within orthopedic cases. A delay in total knee and hip replacements leads to a higher revision and complication rate, reinforcing the significance of early surgical intervention [14]. Institutional and environmental factors relate to this case because strict infection prevention and a lack of surgical delay likely contributed to the successful patient outcome. Early diagnosis and treatment of AVN are crucial to prevent deterioration of the hip joint. There are several operative and non-operative modalities available depending on disease severity. As depicted in this case report, utilizing the appropriate intervention can markedly improve patient outcomes and quality of life.

## Conclusions

This case highlights the successful surgical management of advanced AVN of the femoral head using cementless THA with peripheral screw augmentation and a dual mobility construct. The patient's rapid post-operative recovery, improved range of motion, and pain relief emphasize the effectiveness of this approach in addressing femoral head collapse with poor bone quality. As AVN remains a progressive and debilitating condition, early diagnosis and appropriate intervention are crucial to optimizing patient outcomes. Further research and long-term follow-up studies are warranted to continue refining surgical techniques and improving prosthetic longevity in AVN patients undergoing THA.
